# An Unusual Presentation of Gastric Cancer Metastatic to the Penile Shaft and Perineum

**DOI:** 10.7759/cureus.72924

**Published:** 2024-11-03

**Authors:** Michael G Murray, Athena Masi, Noelle A Kubinak, Lesley A Stead, Thomas R Eanelli

**Affiliations:** 1 Emergency Medicine, MedStar Franklin Square Medical Center, Baltimore, USA; 2 Department of Basic Biomedical Sciences, Touro College of Osteopathic Medicine, Middletown, USA; 3 Medicine, Touro College of Osteopathic Medicine, Middletown, USA; 4 Hematology-Oncology, Goshen Medical Associates, Goshen, USA; 5 Radiation Oncology, Garnet Health, Middletown, USA

**Keywords:** cancer, metastatic gastric adenocarcinoma, penile metastasis, radiation oncoolgy, unusual metastasis

## Abstract

Metastatic disease to the penile shaft is among the most rare metastatic sites for any neoplasm. We report the case of a healthy 69-year-old Jamaican male who initially presented with classical primary gastric adenocarcinoma. He was subsequently treated in a routine manner with complete surgical resection followed by chemoradiation as per the US Intergroup Trial 0116. Two years later, the patient presented with a chief complaint of dyspareunia. He was found to have a perineal nodule and penile skin thickening. He was ultimately diagnosed with gastric metastasis and treated with chemotherapy and salvage radiotherapy. He was followed up for a further 15 months but was eventually lost to follow-up and passed away.

## Introduction

Gastric cancer is the fifth most prevalent neoplasm worldwide and the third most deadly [1], with its subtype adenocarcinoma accounting for approximately 90% of all diagnosed cases. It is a malignant epithelial tumor originating in glandular cells of the stomach. Due to its anatomic location and blood flow, gastric cancer most commonly metastasizes to the liver, peritoneum, and lungs. Unfortunately, survival in metastatic gastric cancer is poor irrespective of staging, site of metastasis, or anatomical location of the cancer [2]. Despite the highly vascular anatomy of the penis, disseminated metastatic cancers to this region are very rare. Only 504 such have been reported worldwide, and the majority of them were in close anatomical proximity to the bladder, prostate, rectum, and sigmoid colon [3]. As of 2015, only three cases of gastric cancer metastasizing to the penis had been reported, constituting only 0.6% of the total cases of primary cancers metastasizing to the penis. In the same report, the main primary cancers that metastasized to the penis were bladder, prostate, and rectal cancer at 29.8%, 29.8%, and 17.3% respectively. These statistics [4] emphasize the rarity of the case we present in this report, which involves gastric cancer metastatic to the penis and perineum, and it is the fourth case to be documented academically [4].

## Case presentation

A 65-year-old Jamaican male presented to his healthcare provider in 2011, complaining of abdominal pain, early satiety, and a 20 lb unintentional weight loss for four to five months. Based on his presentation, an abdominal ultrasound was performed on November 2, 2011, revealing an enlarged pancreatic head (4 cm) but no evidence of intra- or extrahepatic duct dilation. Further evaluation via a CT scan was performed on November 21, 2011, which revealed nonspecific findings incongruent with his complaints. The definitive diagnosis was made through esophagogastroduodenoscopy (EGD) on January 18, 2012, which revealed a large, deep ulcer on the lesser curvature of the gastric antrum. A biopsy from this procedure was consistent with moderately differentiated adenocarcinoma, Helicobacter pylori-negative.

**Figure 1:**
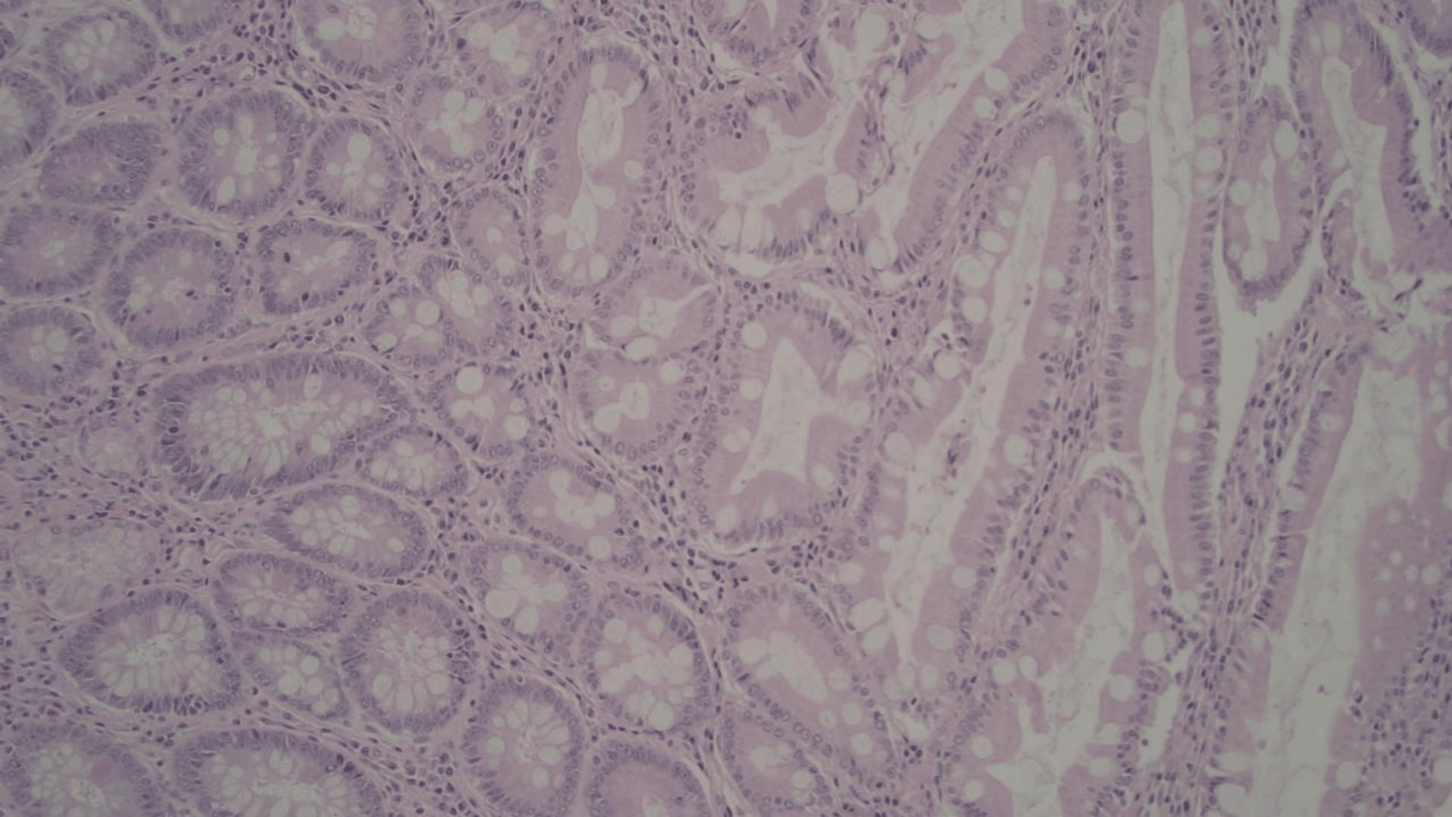
Background gastric mucosa showing chronic gastritis with extensive intestinal metaplasia

**Figure 2:**
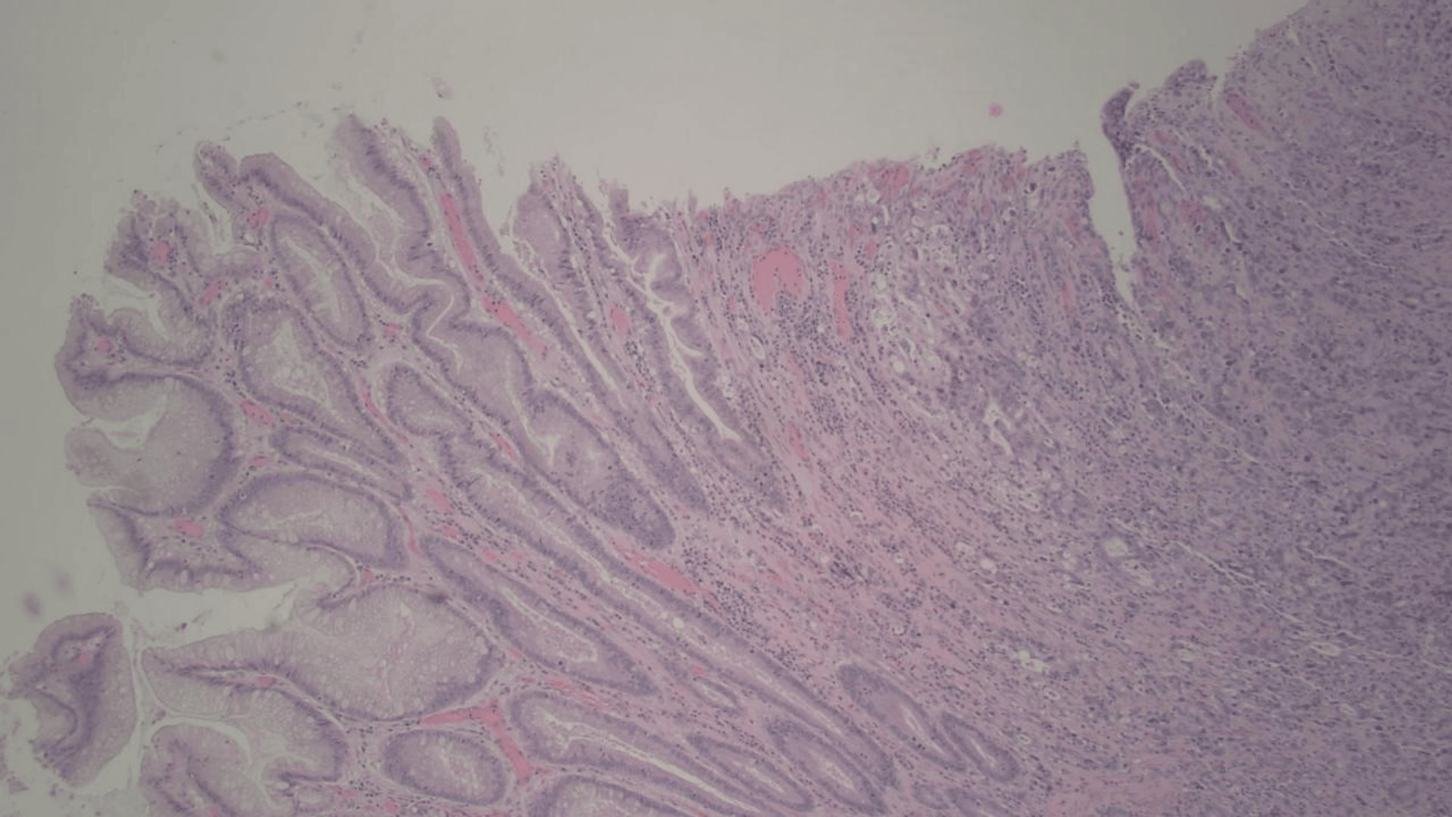
Invasive adenocarcinoma, moderately to poorly differentiated (right) and chronic gastritis with intestinal metaplasia (left)

**Figure 3:**
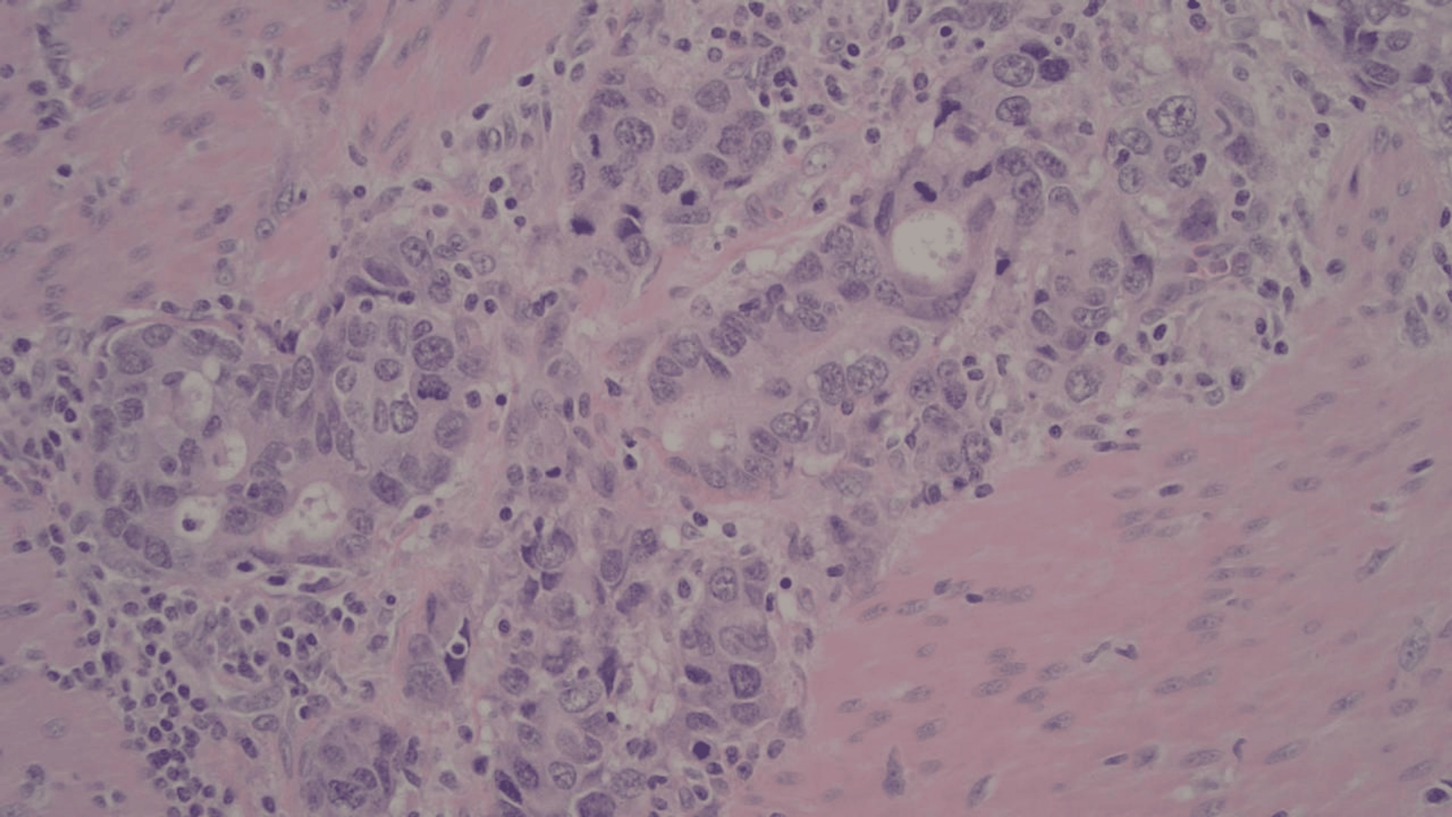
Invasive adenocarcinoma, moderately to poorly differentiated (40x); poorly formed glands, marked cytologic atypia, and numerous mitotes were present

The newly discovered cancer was excised on February 16, 2012, with a final pathology report revealing a poorly differentiated non-mucinous adenocarcinoma measuring 2.5 x 2.5 x 1.4 cm, as seen in Figures 1-3.

**Figure 4:**
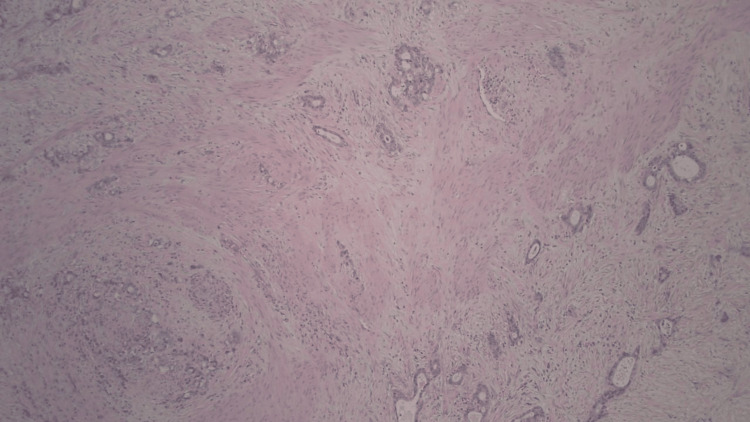
Tumor invading into and through muscularis propria (stage pT3) - image 1

**Figure 5:**
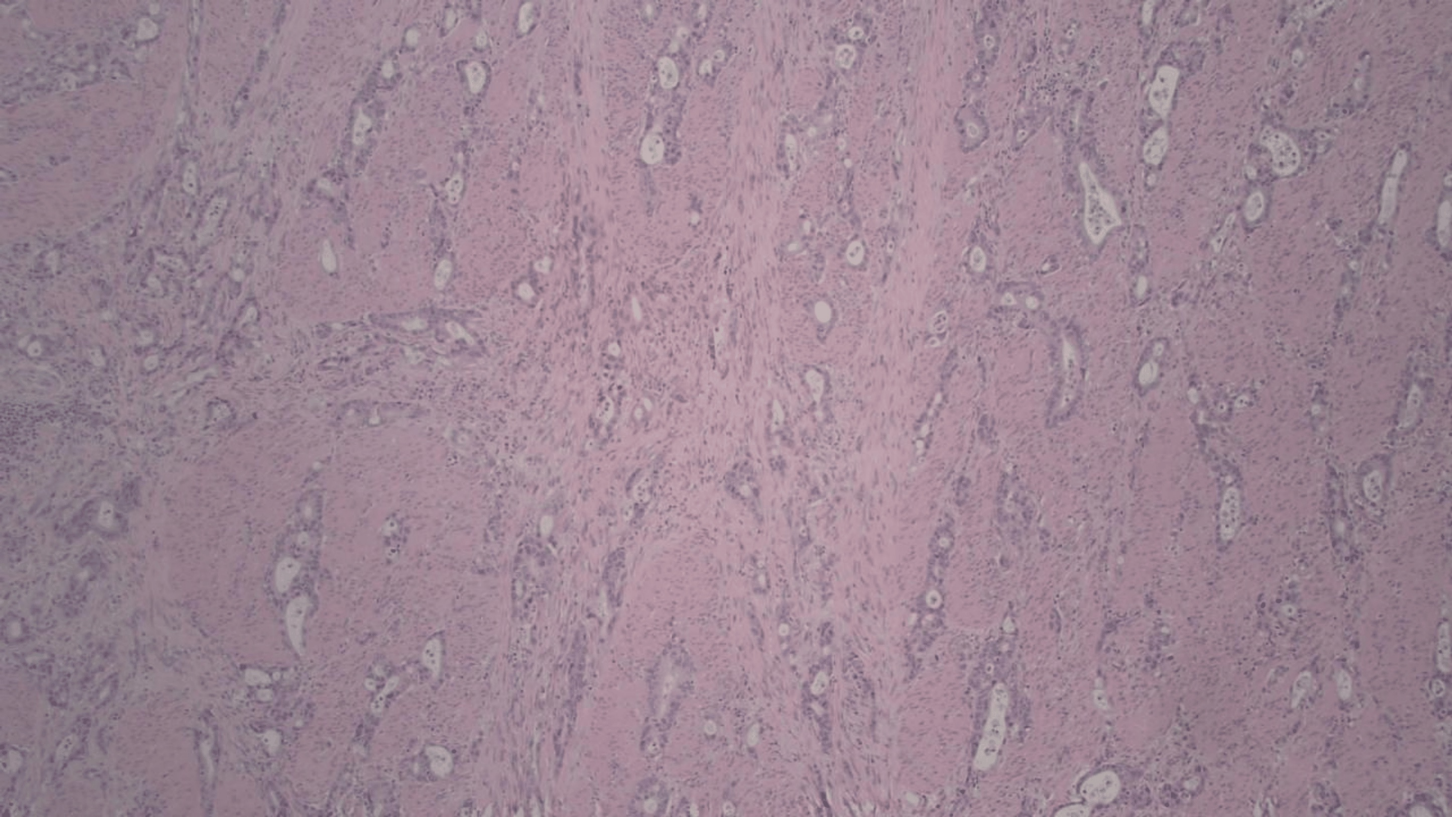
Tumor invading into and through muscularis propria (stage pT3) - image 2

**Figure 6:**
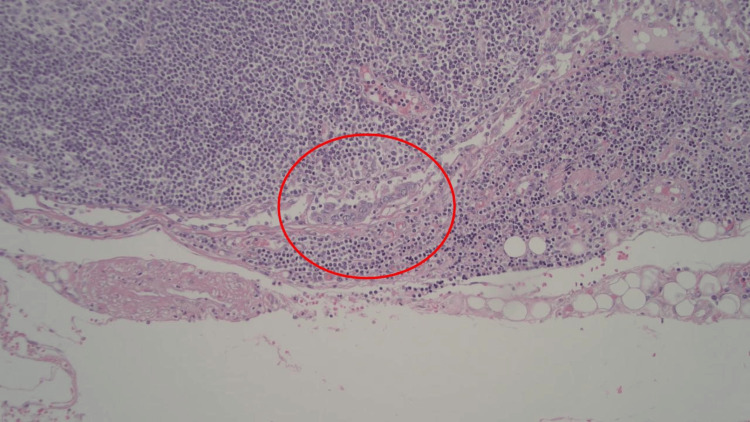
Metastatic carcinoma involving one of four lymph nodes (stage pN1)

**Figure 7:**
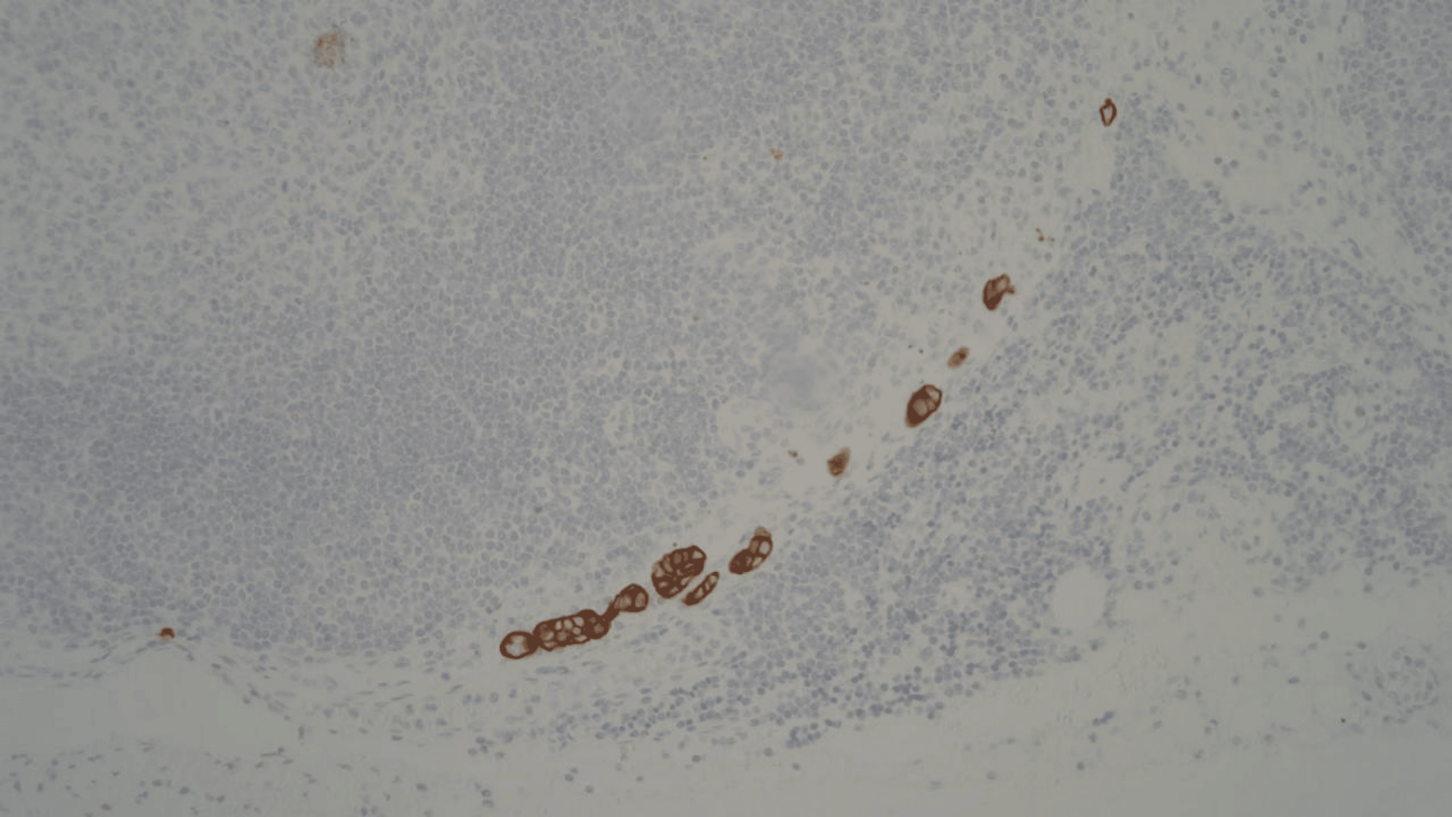
Pancytokeratin stain highlighting metastatic carcinoma in the lymph node

**Figure 8:**
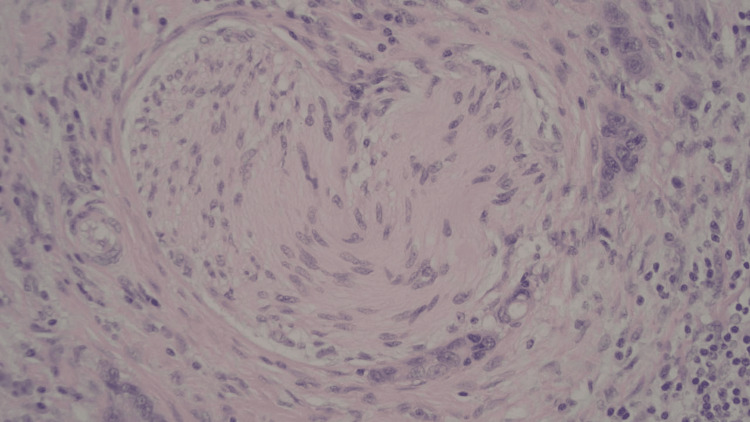
Perineural invasion

Tumor invasion through the gastric wall into the muscularis propria and perigastric adipose was observed; however, there was no invasion into the serosa (Figures 4-5). Margins were all found to be negative. One of four perigastric lymph nodes was positive for metastatic disease (Figures 6-7). Additionally, lymphovascular and perineural spread were noted in pathology (Figure 8). Pathological staging was T3N1M0 stage III adenocarcinoma of the gastric antrum status post subtotal gastrectomy.

The patient received postoperative chemoradiation as per the US Intergroup Trial 0116. This included one cycle of fluorouracil (FU) (425 mg/m^2^ per day) and leucovorin calcium (20 mg/m^2^ per day) daily for five days, followed one month later by 4500 cGy (180 cGy/day) radiotherapy (RT) with FU and leucovorin calcium (400 mg/m^2^ and 20 mg/m^2^ respectively) on days one through four and on the last three days of RT. Two additional five-day cycles of chemotherapy (FU 425 mg/m^2^ per day and leucovorin calcium 20 mg/m2 per day) were given at monthly intervals beginning one month after the completion of RT. The patient tolerated surgery and chemoradiation treatment well and there was no recurrence or metastatic disease.

The patient did not experience any complications in the next two years. However, he presented to medical providers in April 2015 complaining of dyspareunia. A hard nodule was noted in the perineum on physical examination. The most notable clinical manifestations of penile metastasis range from heterogeneous penile nodules to masses, with or without ulceration, obstructive or irritative urinary symptoms, hematuria, or priapism. An MRI without contrast was performed on April 14, 2015, which showed an enlarged heterogeneous prostate (as previously detected three years prior) with T2 darkening and mild enhancement, as well as thickened skin in the perineum.

A testicular sonogram was done for further evaluation due to the noted perineal nodule and elevated AFP levels. The evaluation revealed appropriately sized testicles with a prominent rete testis or mass with calcification and shadowing in the left testicle measuring 1.5 x 1.1 x 1.2 cm. Subsequently, skin thickening was noted along the penile shaft, and a penile shaft biopsy was taken on April 30, 2015, revealing gastric tumor cells positive for CK7, P16, and calretinin, but negative for CK20, PSA, PSAP, TTF-1, and PAX8. This immunohistochemical profile was critical in confirming the metastatic origin from the stomach, as the markers CK7 and P16 are commonly expressed in gastric adenocarcinomas, while the absence of markers like PSA and PSAP rules out prostate cancer. The previous gastrectomy specimen was concurrently reviewed, and both tumors revealed identical morphology. The penile shaft biopsy was thus found to reveal metastatic adenocarcinoma consistent with gastric primary.

The patient began salvage chemotherapy with a regimen consisting of epirubicin, oxaliplatin, and Xeloda. A clinical exam after one cycle of this regimen revealed softening of the penile lesion. After the completion of his first chemotherapy cycle, the patient was admitted to the hospital with a diagnosis of gastric outlet obstruction. Again, a biopsy was taken, which showed no evidence of gastric recurrence but confirmed a functional gastric outlet obstruction. Due to the patient's unrelated health complications and difficulty in tolerating salvage chemotherapy, it was decided to proceed with salvage RT of the penile shaft and perineal area instead of beginning a new cycle of chemotherapy.

Upon presenting for RT, his penis, perineum, and testicles were unremarkable on a clinical exam, demonstrating a complete response to his first chemotherapy trial. Given the often poor prognosis of these tumors, the therapeutic strategy was essentially oriented towards organ preservation and improved quality of life. Palliative care is key in cases of metastatic cancer, where the goal shifts from curative treatment to maintaining the patient's quality of life and minimizing invasive procedures. The decision was made to monitor the patient closely without RT. Unfortunately, a short time into the monitoring period, penile skin thickening was noted, inguinal nodes were palpable, and lesions were palpated on the testicles and perineum. Hence, RT had to be initiated.

At the time of submission of this case report, the patient had confirmed gastric cancer metastatic to the penile shaft for 15 months. He was subsequently followed up closely to monitor for additional metastatic lesions, none of which were identified. The patient was eventually lost to follow-up and has since passed away. The patient's relatively prolonged survival following the diagnosis of penile metastasis is noteworthy, as most patients with such disseminated disease have a much shorter survival time.

## Discussion

The rarity of our case is illustrated in the statistics presented in Table 1, which provides a summary of the primary sites of penile metastasis in published reports.

**Table 1:** Summary of primary sites of penile metastasis in published reports* *[4]

Primary cancer	Cases reported until 2015	Percentage of total cases
Genitourinary	335	69.8
Bladder	143	29.8
Prostate	143	29.8
Kidney	31	6.5
Testis	12	2.5
Other	6	1.2
Gastrointestinal	101	21.1
Colon/rectum	83	17.3
Hepatobiliary/pancreas	8	1.7
Esophagus	5	1
Stomach	3	0.6
Other	2	0.4
Respiratory	26	5.4
Lung	20	4.2
Upper airway	6	1.3
Bone	3	0.6
Others	15	3.1
Total	480	100

Secondary neoplasms to the penis and perineum are an extremely rare phenomenon, and their exact incidence rate is not known due to the lack of studies and presentations. This entity has been academically reported 504 times [3,4] since the earliest known report in 1870 by Eberth [5]. Of these cases, most of the primary cancers have been in close anatomical proximity to the prostate, rectum, sigmoid colon, and bladder [6]. Given this evidence, there is an anatomical component to penile spread. Mechanisms related to penile spread were originally described by Paquin [7] in 1956; however, given the paucity of penile metastasis and the fact that patients usually present with end-stage disseminated disease, it is likely that multiple mechanisms play a role, which has to be analyzed on a case-by-case basis. Another unique feature of this particular case was the manner in which the metastasis manifested. This involved a primary recurrence of gastric cancer after lacking clinical symptomatology for approximately two years, which is a far cry from the widespread disseminated disease typical of this type of presentation. Given this fact, our patient’s survival is among the longest recorded in the current literature after penile metastasis was found at approximately 15 months.

Based on Paquin’s original study and current literature, the route of metastasis can be classified into the following categories: retrograde venous spread, retrograde lymphatic spread, arterial spread, direct tumor invasion, and iatrogenic implantation [8]. It is important to bear in mind that since we are discussing a wide variety of primary tumors, each has a preferred mode of metastasis. In general, each mechanism may play a role in a particular case. We will briefly discuss this and present the most likely means of metastasis in our case. The most logical one is direct tumor invasion (contiguous spread) as the majority of penile metastasis occurs from areas in close proximity, such as the aforementioned bladder, prostate, and recto-sigmoid colon. Curiously, however, this does not seem to play a major role in penile spread as penile metastasis is rarely found at the base of the penile shaft. Intuitively, direct tumor invasion can be confidently ruled out as a means of metastasis in our case.

Given the large proportional blood flow to the penis relative to anatomical size, the arterial spread seems like a reasonable explanation for metastatic spread, especially since during a given night tumescence can occur an average of four to five times during which over 100 mL of blood can be retained for significant periods [9]. Traditional hematogenous spread is seen more frequently in sarcomas as opposed to carcinomas, our patient's presentation. Given the amount of blood flow, it seems odd that overall metastatic rates are as uncommon as observed clinically. It is possible that cancer cells are seeded to the penile arterial network; however, the conditions are not appropriate for cells to thrive.

Retrograde venous and retrograde lymphatic spread are the traditional routes of metastatic spread for carcinomas and are the most likely ones in this patient, as well as in the majority of penile metastasis cases. There is a complex but well-established communication between the penile venous plexus and the pelvic venous plexus; this, compounded with the highest rate of penile metastasis occurring in the corpora cavernosa [10], the location in greatest communication with venous blood, makes venous spread the most likely phenomenon in our case as well as the most frequent mode of spread. It is important to note that the original pathology report noted lymphovascular spread. Retrograde venous and lymphatic spread near the tumor is quite plausible due to the likely blockage of the venous and lymphatic network; however, the transit from the epigastric venous network to the pudendal venous network is more difficult to explain.

## Conclusions

We discussed a case of primary gastric cancer with metachronous metastasis to the penis and perineum approximately two years after its original diagnosis and treatment. Such a presentation has been documented only three times in the literature previously and is a unique presentation of a common primary tumor, which is of great significance academically. Clinicians should ensure that metastatic disease is ruled out in patients presenting with penile pain and dyspareunia after prior treatment for any type of neoplastic condition.
